# Wastes from Agricultural Silage Film Recycling Line as a Potential Polymer Materials

**DOI:** 10.3390/polym13091383

**Published:** 2021-04-23

**Authors:** Jerzy Korol, Aleksander Hejna, Klaudiusz Wypiór, Krzysztof Mijalski, Ewelina Chmielnicka

**Affiliations:** 1 Department of Material Engineering, Central Mining Institute, Pl. Gwarków 1, 40-166 Katowice, Poland; kwypior@gig.eu (K.W.); kmijalski@gig.eu (K.M.); 2 Department of Polymer Technology, Gdansk University of Technology, Narutowicza 11/12, 80-233 Gdansk, Poland; aleksander.hejna@pg.edu.pl; 3 Paint & Plastics Department in Gliwice, Institute for Engineering of Polymer Materials and Dyes, 50 A Chorzowska Street, 44-100 Gliwice, Poland; e.chmielnicka@impib.pl

**Keywords:** recycling, agricultural film, polymer composites, polyethylene, compatibilization

## Abstract

The recycling of plastics is currently one of the most significant industrial challenges. Due to the enormous amounts of plastic wastes generated by various industry branches, it is essential to look for potential methods for their utilization. In the presented work, we investigated the recycling potential of wastes originated from the agricultural films recycling line. Their structure and properties were analyzed, and they were modified with 2.5 wt % of commercially available compatibilizers. The mechanical and thermal performance of modified wastes were evaluated by tensile tests, thermogravimetric analysis, and differential scanning calorimetry. It was found that incorporation of such a small amount of modifiers may overcome the drawbacks caused by the presence of impurities. The incorporation of maleic anhydride-grafted compounds enhanced the tensile strength of wastes by 13–25%. The use of more ductile compatibilizers—ethylene-vinyl acetate and paraffin increased the elongation at break by 55–64%. The presence of compatibilizers also reduced the stiffness of materials resulting from the presence of solid particles. It was particularly emphasized for styrene-ethylene-butadiene-styrene and ethylene-vinyl acetate copolymers, which caused up to a 20% drop of Young’s modulus. Such effects may facilitate the further applications of analyzed wastes, e.g., in polymer film production. Thermal performance was only slightly affected by compatibilization. It caused a slight reduction in polyethylene melting temperatures (up to 2.8 °C) and crystallinity degree (up to 16%). For more contaminated materials, the addition of compatibilizers caused a minor reduction in the decomposition onset (up to 6 °C). At the same time, for the waste after three washing cycles, thermal stability was improved. Moreover, depending on the desired properties and application, materials do not have to go through the whole recycling line, simplifying the process, reducing energy and water consumption. The presented results indicate that it is possible to efficiently use the materials, which do not have to undergo the whole recycling process. Despite the presence of impurities, they could be applied in the manufacturing of products which do not require exceptional mechanical performance.

## 1. Introduction

The use of plastics in the agriculture industry is very high because of their useful properties and relatively low price [1]. For the preservation of fodder by ensilage, mostly polyethylene (PE) films are used, mainly low-density polyethylene (LDPE). It is a low-cost material that can be easily processed into products meeting the criteria related to agricultural films’ optical and physical properties [2]. In southern European countries, plastics are mainly used for crop protection (in greenhouses, cultivated tunnels, mulching). It is associated with unfavorable climate conditions, so films are applied to improve the microclimate for crop growth [3]. In contrast, in the northern countries, these materials cover the pass-through silos, wrap bales, and the latest trend in agricultural production: maintenance in foil sleeves [4]. The requirement for a conditional ensilage process is fast and accurate sealing of the ensiled plant price against weathering tests, and ensiled plant material is tightly covered repeatedly wrapped with a layer of foil or closed in a foil sleeve [5]. The unique advantages of silage production from foil are small losses in nutrients (law, energy), low dependence of farmers on weather conditions, quick supplementation of conventional feed, and low storage costs [6,7,8]. Nowadays, the ensilage film used is made of polyethylene resistant to biotic and abiotic factors. After the usage, it becomes a waste product, and its decomposition takes many years, with the release of many harmful compounds into the environment [9,10,11]. The foil used to cover the silage stocks could be easily reused. However, it is difficult to reuse it in agricultural activities. Films used for wrapping bales with hay or the one from foil sleeves are often cut discontinuously, limiting their reuse in primary applications. Agricultural plastics are contaminated with earth, sand, dirt, biomass, and moisture [12,13,14]. Moreover, residual organic chemicals such as fertilizers, herbicides, and pesticides may be present, which can be harmful to the environment [15]. Moreover, microorganisms developing on their surface (lactic acid bacteria, the organisms behind its production, which are necessary to take care of the ensilage course) cause a strong unpleasant smell. Currently, the agricultural films’ recycling rate is estimated as not exceeding 30% [16]. Such a phenomenon is also attributed to the relatively high cost of reusing agricultural films [17]. After primary use, silage foil is often used as waste on legal and illegal waste landfills or burned in fields or in home boiler rooms [18]. Such disposal is harmful to the environment and is a serious issue because plastic waste may accumulate in natural ecosystems [19]. Plastic materials undergo to fragmentation, facilitating their dispersion in the environment and making their collection nearly impossible [20]. Such fragmentation also enables the release of additives used in plastics production, e.g., antioxidants, dyes, plasticizers, or stabilizers, which can accumulate in the environment [21]. As a result, microplastics affect the quality of natural waters and soil [22,23]. According to the literature data [24], the global use of plastics in agriculture exceeds 6 MT annually and, by 2030, this value could increase by 50% due to the growing demand of the increasing population. Currently, LDPE films alone account for over 2 MT of agricultural plastics [3]. Considering the contaminations mentioned above, such as soil and organic matter, post-consumer agricultural plastics would account for ~ 17 MT of waste. Therefore, it is crucial to develop the processes allowing the recycling of agricultural films, possibly for other applications. Nevertheless, such a solution requires appropriate adaptation of the recycling processes allowing the processing of agricultural films [25]. As mentioned above, the biggest issue is the complex nature of the waste stream in plastic compositions and contaminants [26]. Their presence may noticeably affect the material’s mechanical and thermal performance, significantly limiting the potential application range. The presence of impurities decreases the homogeneity of material. It reduces its cohesion due to the often weak interfacial interactions between the hydrophobic polyolefin matrix and solid particles of soil or lignocellulosic materials [27]. Multiple research works [28,29,30] indicated the significant differences in the polarity between plant-based fillers and nonpolar polyolefin matrix. Such an effect can be attributed to the presence of multiple functional groups, mostly hydroxyls, on the presence of lignocellulosic materials [31]. Most mineral fillers also show hydrophilic character and have high surface energy, limiting the interfacial adhesion with polymer matrices [32]. Therefore, it is crucial to modify the waste streams in the possibly most straightforward processes with low environmental impact. One of the simplest approaches is the incorporation of compatibilizers, which aim to enhance the interfacial interactions in composite materials, simultaneously overcoming the drawbacks originating from the presence of impurities [33]. Moreover, compatibilizers may also improve the interactions between different polymers present in waste materials originated from plastic recycling [34]. Strong interfacial interactions are essential to obtain materials with satisfactory mechanical performance. Such an approach may be realized by providing chemical bonding possibilities between the polymer matrix and impurities, which can be treated as fillers [35]. By far the most popular compatibilizers, which have been commercially available for many years, are polyolefins functionalized with maleic anhydride (MA). They are miscible with the most popular polymers—polyethylene (PE) and polypropylene (PP) [36,37]. Their popularity is associated with maleic anhydride’s ability to react with the most popular functional groups present on fillers’ surface—hydroxyl groups. Such compounds may significantly enhance recycled polymers’ performance, containing multiple impurities improving the stress transfer at the interface and interfacial adhesion, inhibiting delamination of material [35]. As a result, the mechanical properties of waste streams are improved, and materials are characterized by lower stiffness, increased tensile strength, or elongation at break. Such changes may noticeably enhance their application potential in products that do not require exceptional mechanical performance.

This paper aims to investigate the recycling potential of various wastes from the agricultural films’ recycling line. To enhance their performance and provide the potential applications of waste-based materials, various compatibilizers commonly present on the market were applied. The mechanical and thermal properties of the new waste-based materials were analyzed and discussed. The potential application of the analyzed wastes after compatibilization would significantly shorten and facilitate the recycling process, allowing the skipping of the compaction, grinding, and extrusion phases. As a result, the potential application window of the wastes could be significantly broadened. They could be implemented as raw materials in other processes resulting in products based on polymer materials, which do not require exceptional mechanical performance. Such products could be obtained from raw materials generated with reduced amounts of energy and water, compared to the regranulate obtained after the complete recycling process. It could noticeably enhance their environmental friendliness and reduce the environmental burdens of final products, which is essential considering current trends and permanently tightening regulations.

## 2. Materials and Methods

### 2.1. Materials

**Figure 1:**
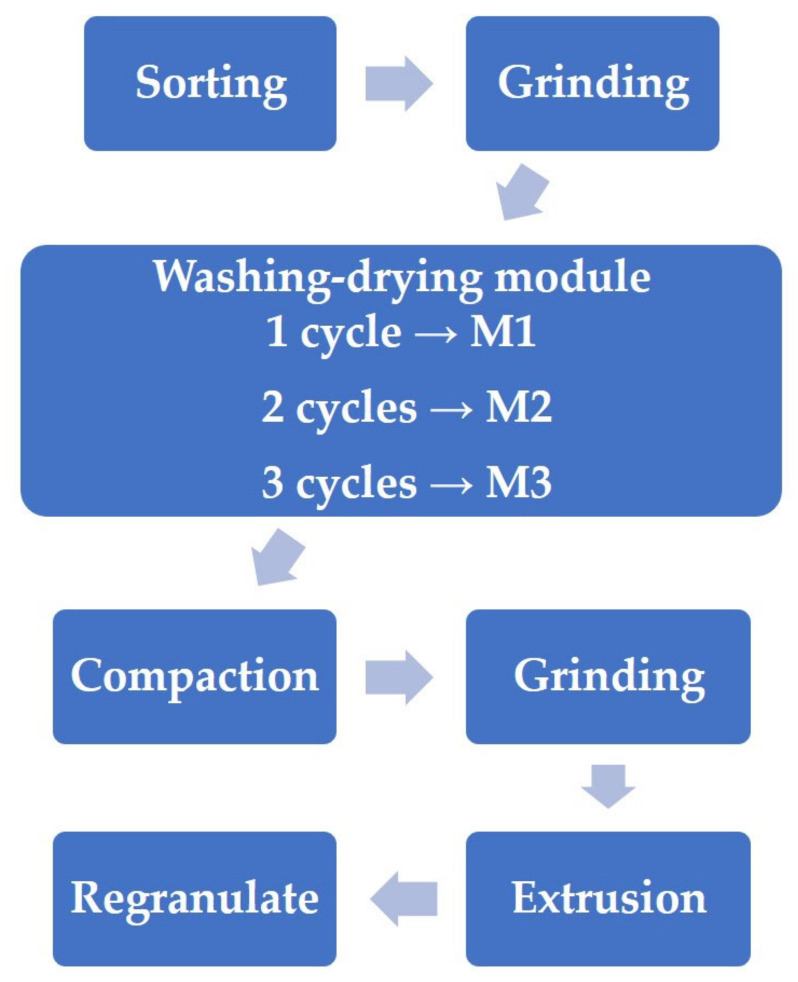
General scheme of the recycling line, from which wastes were collected.

**Figure 2:**
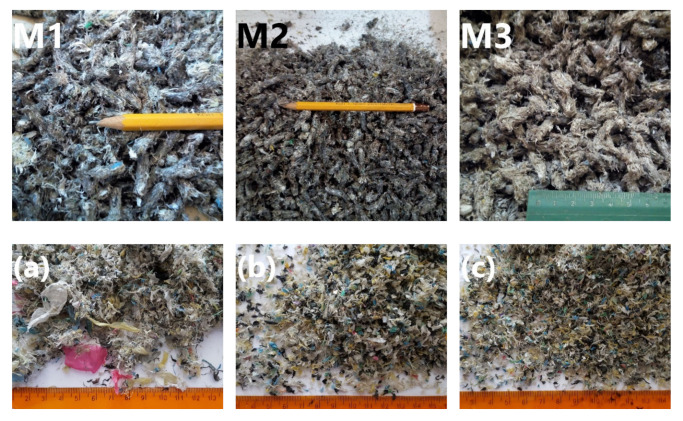

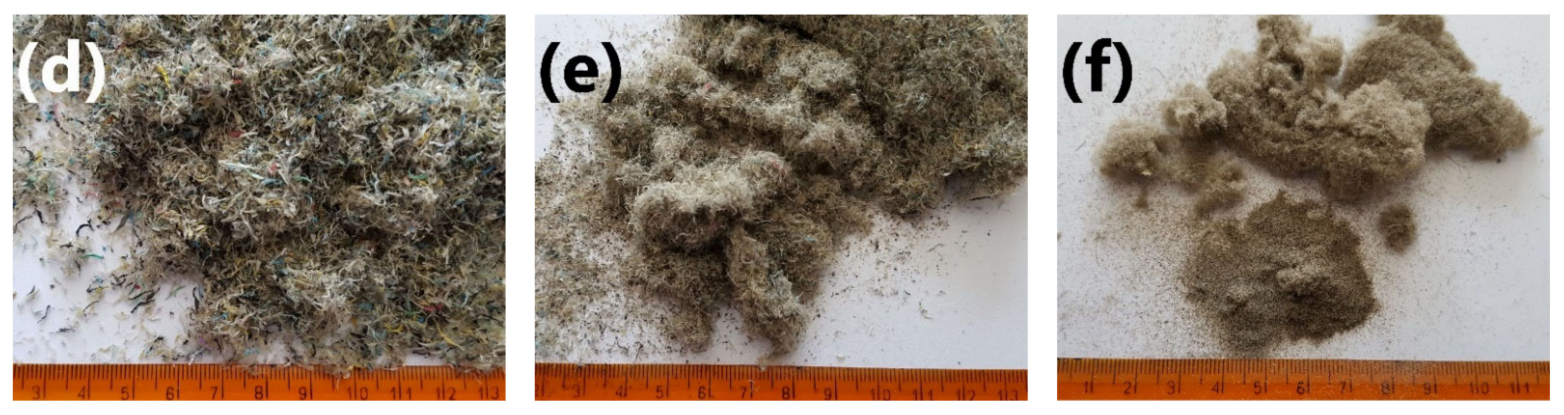
The appearance of M1, M2, M3 wastes and impurities removed during washing and retained on sieves with (**a**) 4 mm, (**b**) 2 mm, (**c**) 1 mm, (**d**) 0.5 mm, (**e**) 0.125 mm, and (**f**) residue.

The main materials used in this work are wastes from the recycling line for waste agricultural silage film from a local recycling company (Bieruń, Poland). The general scheme of the recycling line is presented in Figure 1. These recycled agricultural polymer films are based mainly on low-density polyethylene but may contain high-density polyethylene (HDPE) and polypropylene residues. There are three wastes analyzed in this work, which come from consecutive dryers after washing on high-speed dynamic washing baths (M1, M2, M3). The washing process is performed with water, without any addition of detergents or other surfactants. Such an approach should be considered environmentally friendly because it reduces the additional emissions of chemicals to the environment and reduces the extent of plastic decomposition during recycling [38,39]. Sample M1 is a waste material after the first washing-drying cycle, M2 after the second, and M3 after the third. Samples M1–M3 were obtained as regrinds. Figure 2 presents the appearance of applied wastes and impurities removed during the washing process.

As mentioned above, wastes applied as the polymer matrix in the presented work were generated during agricultural films’ recycling. Therefore, they contained mineral and organic matter residues, which are often not compatible with the polyethylene matrix. Therefore, to enhance prepared materials’ performance, different compatibilizers based on polyethylene, polypropylene, ethylene-vinyl acetate, styrene-ethylene/butylene block copolymers, or paraffin, as listed in Table 1, were applied.

### 2.2. Sample Preparation

All samples were prepared using a twin-screw extruder Leistritz ZSE model 27 HP (Nuremberg, Germany). The screw diameter was 27 mm, and the length to diameter ratio was 44 (L/D = 44). The extruder had ten heating and cooling zones. Constructions of the used plasticizing systems and detailed descriptions of screws are shown in previous work [40]. Screws consisted of feeding, plasticization, degassing, mixing, degassing, mixing, degassing, and dosing zones. The length of the plasticization zone equaled 4.4D, and the screws consisted of kneading segments with a varying angle between the particular cam disks (30°, 60°, and 90°). The increase in the angle between the cam discs changes the segment character from transportation to mixing and shearing. The length of the first and second mixing zones equaled 3.3D and 2.2D. They consisted of the same kneading elements as the plasticization zone. All materials were dosed by Brabender gravimetric screw feeders (Duisburg, Germany) with a constant flow rate of 20–22 kg/h. The extruder barrel’s temperature was set at 140–180 °C and screw speed at 250–300 rpm.

Obtained extrudates were injection molded using Arburg type Allrounder 270-210-500 injection-molding machine (Loßburg, Germany) into standard so-called dog–bone specimen type 1A (ASTM 527) with the cross-section of the measurement part equal to 40 mm^2^; width of the narrow parallel-sided portion of 10 mm; width at ends of 20 mm; thickness of 4 mm; gauge length of 80 mm; length of narrow parallel-sided portion of 109.3 mm; overall length of 170 mm. The machine is equipped with a Priamus (Schaffhausen, Switzerland) injection process controller. Sample injection parameters: temperature of the polymer melt—120 °C ± 2 °C, form temperature—20 °C ± 1 °C, injection speed—190 mm/s, cycle period—60 s, injection pressure—650 bar, clamping pressure—350 bar.

Except for the unmodified M1–M3 samples, compatibilized variants were prepared. For all waste streams, the compatibilizers were introduced in the amount of 2.5 wt % based on our previous experience.

### 2.3. Measurements

The particle size distribution of samples M1–M3 was determined according to PN-EN 933-1 standard using a LPzE-4e siever from MULTISERW-Morek Jan Morek (Brzeźnica, Poland) with sieves characterized by following openings: 0.125, 0.5, 1, 2 and 4 mm. For each material, three 500 g samples were analyzed.

The FTIR spectra were recorded on an FTIR Nicolet 380 spectrometer from Thermo Fisher Scientific (Waltham, MA, USA) in reflectance mode. Reflectance infrared spectra were measured using an attenuated total reflectance (ATR) technique. The spectrometer was equipped with a DTGS detector and KBr beam splitter. All spectra in the wavenumber range 350–4000 cm^−1^ were recorded with a spectral resolution of 2 cm^−1^ and were averaged over 64 scans. For each material, three spectra were recorded.

Thermal properties, particularly the crystallization and melting behavior, were investigated using Mettler Toledo TGA/DSC 1 instrument (Columbus, OH, USA). The samples were first heated to 180 °C at 10 °C/min and then held isothermally for 2 min. After that, the samples were cooled at −10 °C/min and held isothermally for 2 min. These steps were all run once and then again. After the first cooling and then reheating, the stress relief and enthalpic relaxation effects were eliminated, such as the samples’ thermal history. Thermal properties, like melting and crystallization temperatures and enthalpies, were determined from the second scans. For each material, at least two samples were analyzed.

The thermal stability of analyzed materials was studied by thermogravimetric analysis (TGA) with a Mettler Toledo TGA/DSC 1 instrument (Columbus, OH, USA). The sample weight was about 15 mg, and the heating rate was 10 °C/min in the temperature range from 25 to 900 °C in the air atmosphere. For each material, at least two samples were analyzed.

Strength properties were estimated by determining the elastic modulus, tensile strength, and elongation at break. The tests were carried out based on the PN-EN ISO 527 standard using the Instron (Norwood, MA, USA) strength testing machine with an elongation head and an extensometer. The elongation velocity was 1 mm/min for measuring the elastic modulus and 50 mm/min to measure tensile strength and elongation at break. For each material, at least five samples were analyzed.

## 3. Results

### 3.1. Characterization of Applied Wastes

**Figure 3:**
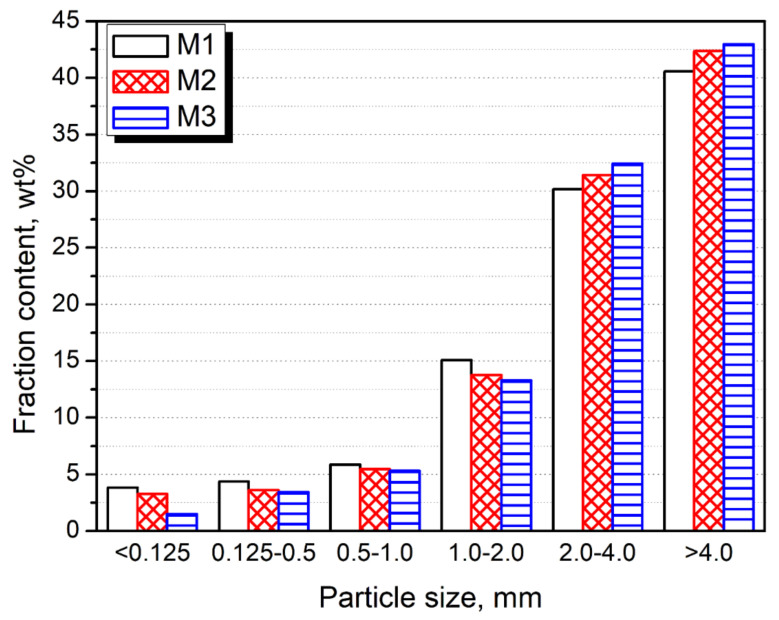
The particle size distribution of analyzed wastes.

Figure 3 shows the particle size distribution of investigated wastes. It was found that the highest content of the smallest particles with diameters below 0.5 mm was noted for the M1 sample, while the lowest was noted for the M3 sample. Such an effect was particularly pronounced for diameters lower than 0.125 mm. Except for the smallest regrind of polymer waste, these fractions contained a significant portion of impurities, especially the mineral ones—residual minerals, soil, or sand (see Figure 2). Therefore, it can be found to be very beneficial that each washing cycle reduced the smallest particles’ content, pointing to contaminants’ removal. Nevertheless, they were not completely removed even after three washing cycles, indicating that they are strongly bound with the polymer materials. The presence of impurities, even in the M3 sample, was later confirmed by the FTIR analysis.

**Figure 4:**
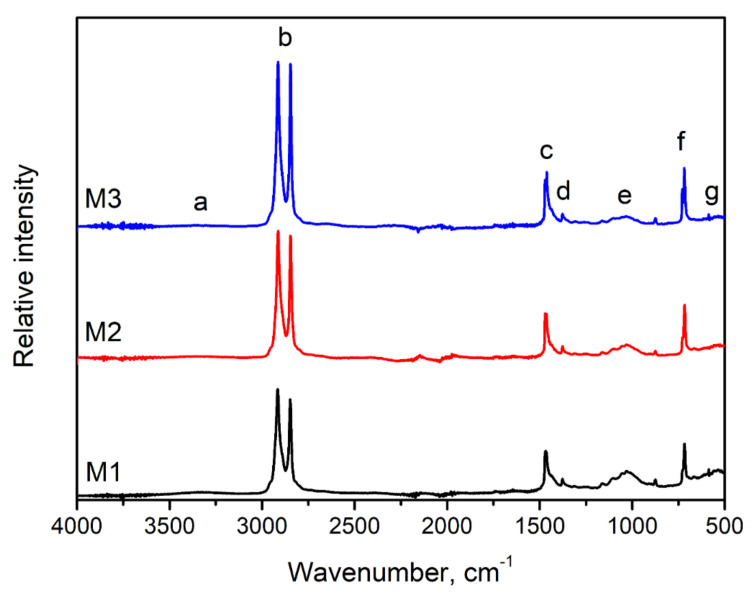
Fourier-transform infrared spectra of analyzed wastes (letters a–g indicate the particular signals described in the text).

In Figure 4, there are FTIR spectra of the applied M1-M3 samples presented. It can be seen that the obtained spectra are typical for the polyolefins [41]. The intensive absorption bands (b) at 2847 and 2914 cm^−1^ were attributed to the symmetric and asymmetric stretching vibrations of C-H bonds. Moreover, a small bump at 2950 cm^−1^ can be noted, which may point to the polyethylene contamination with a small portion of polypropylene. Peaks related to the bending deformations of C-H bonds (c) were noted around 1460 cm^−1^, while signals (f) around 718 and 730 cm^−1^ were associated with the rocking vibrations of the PE macromolecule. The presence of these signals is strictly related to the chemical structure of polyethylene. Their intensity was increased after washing, which points to an efficient washing process.

**Figure 5:**
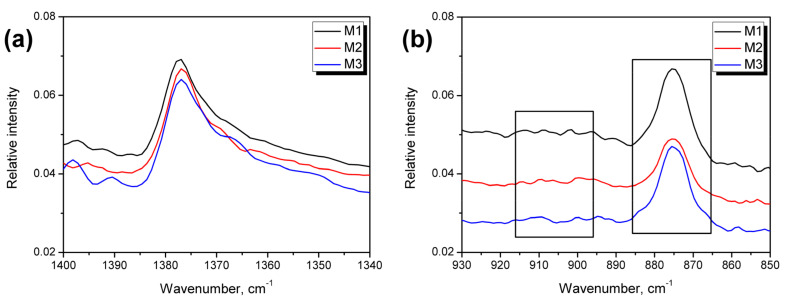
Detailed Fourier-transform infrared spectra of analyzed wastes in the range (**a**) 1340–1400 cm^−1^ and (**b**) 850–930 cm^−1^.

Another signal characteristic of polyethylene is signal (d) noted around 1377 cm^−1^, also presented in Figure 5a. The wavenumber range from 1340 to 1400 cm^−1^ may be used to identify the primary type of polyethylene material, according to Jung et al. [42]. They indicated that the presence of the most significant signal at 1377 cm^−1^, with an only minor but visible signal at 1368 cm^−1^, points to the highest share of low-density polyethylene or its linear type, which is in line with the information provided by the recycling company. Nevertheless, traces of the HDPE can also be present, later evaluated by the differential scanning calorimetry. Such an effect was also earlier described by Gulmine et al. [43]. According to Nihikida and Coates [44], the LDPE and LLDPE may be distinguished by analyzing the 650–1000 cm^−1^ region, particularly signals around 900 cm^−1^. The Authors reported that for the linear type of LDPE, the bands marked in Figure 5b are equally weak, while for LDPE, the one at lower wavenumbers is noticeably larger. Such an effect is observed for the analyzed wastes, which confirm that the share of a linear variant of low-density polyethylene is noticeably lower than those of LDPE.

However, differences between samples were noted, which was associated with the recycling line’s efficiency and the presence of contaminations. The seven main signals or groups of signals were detected. In the range of 3200–3500 cm^−1^, the broad and small signals (a) were noted, related to O-H and N-H bonds’ stretching vibrations. The effect was most pronounced for the M1 sample due to the organic contaminants’ presence in waste material, such as silage or plant residues. Other peaks (e) associated with the organic impurities were detected in the range of 1000–1160 cm^−1^. They were characterized for the different types of vibrations of C-C, C-H, C-O, and C-O-C bonds present in the structure of lignocellulosic materials [45]. Moreover, peaks around 1000 cm^−1^ may also indicate the residual polypropylene presence [42].

Except for the plant-based impurities, agricultural films may also contain soil residues. Signals associated with the inorganic materials are mostly observed below 1200 cm^−1^. Signals (e) around 1000–1160 cm^−1^, except for lignocellulosic materials, are also characteristic for kaolinites, Si-O, and O-Si-O bonds in clays and quartz [46]. Additionally, multiple bands (g) below 700 cm^−1^ were attributed to quartz, dolomite, and calcite, showing the presence of Ca and Mg, which are often present in soil [47]. Despite these signals’ low magnitude, it significantly decreased for sample M3, which indicated the washing process’ good efficiency.

For a more detailed analysis of the chemical structure of M1–M3 wastes, in Table 2 there are presented absorbance values at particular wavenumbers, which were used to calculate the carbonyl index (CI) according to the literature data [48,49,50,51,52,53]. Carbonyl index is calculated as the ratio of peaks’ height at particular wavenumbers or areas of particular peaks. It can be seen that, despite the additional washing–drying cycles, the values of the carbonyl index are decreasing. Such an effect is attributed to the efficiency of the washing process and decreasing impurities content, which may contain the C-O-C structures. According to literature reports [54,55,56] it may affect the calculated values of the carbonyl index. Other factors, which have an impact on its value may be sample thickness, mode of performed FTIR analysis, resolution of analysis, and quality of the equipment [51]. Almond et al. [51] suggest that area-based calculation methods are more precise because they are based on a broader data range than only particular points. Nevertheless, it can be seen that, independently of the selected method for CI calculation, its values are decreasing with the number of applied washing cycles. Such an effect suggests that the presence of impurities has a very significant impact on the calculations. Moreover, it could be concluded that the oxidative degradation of the polymer phase does not occur during the washing–drying step of the recycling process.

**Figure 6:**
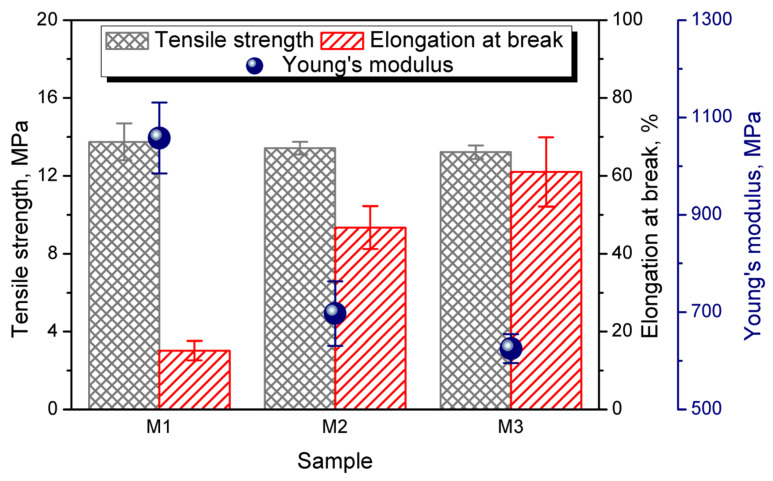
Tensile strength, elongation at break, and Young’s modulus of analyzed wastes.

Figure 6 presents the results of static tensile tests performed for applied wastes M1–M3. It can be seen that all samples were characterized by similar values of tensile strength, irrespective of to the above-mentioned impurities. Nevertheless, despite their low content, solid particles of organic and mineral residues significantly affect the homogeneity of materials, expressed by the noticeable enhancement of elongation at break after washing from the initial 15 to 61% for the M3 material. Such an effect points to the effectiveness of the washing process. However, these values are still noticeably lower than typical literature values, pointing to the presence of residual contaminants [57]. Moreover, due to the enhanced ductility of materials, a significant drop in stiffness was noted.

**Figure 7:**
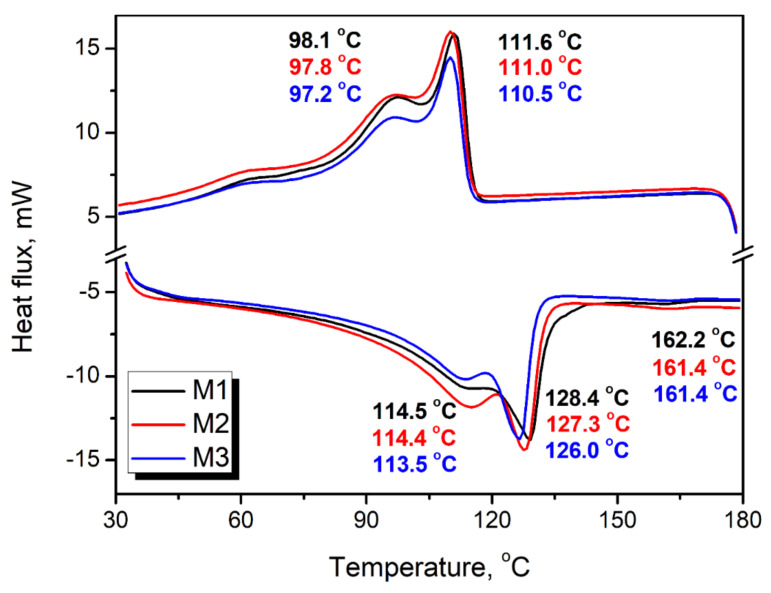
Cooling (**upper**) and heating (**lower**) thermograms (presented in “exo up” mode) of analyzed wastes obtained with differential scanning calorimetry.

Figure 7 presents the results of the differential scanning calorimetry analysis of the applied polyethylene wastes. During heating of materials, three endothermic peaks, attributed to melting of particular components of samples at temperatures around 113.5–114.5, 126.0–128.4, and 161.4–162.6 °C, respectively, were noted that correspond to the melting of LDPE, HDPE, and PP [58]. The peak associated with the polypropylene showed shallow magnitude, with the ΔH_m_ in the range from −0.73 to −0.84 J/g. Therefore, considering that typically values of 80–90 J/g were reported in the literature [59], obtained results indicate very low PP content in presented materials, which confirm the FTIR results. Peaks attributed to LDPE and HDPE’s presence were noticeably more robust and indicated the presence of both polyethylene types in the analyzed waste streams [60]. The above-mentioned spectroscopic analysis results pointed to the significantly higher share for the low-density type in M1–M3 samples, confirmed by DSC measurement. Based on the literature data and the shape of obtained thermograms, it can be stated that the share of LDPE in the whole polyethylene phase of waste exceeds 85% [60,61]. The sharpening of the peaks for the M3 compared to M1, especially on the heating curve, points to the increased homogeneity of the material, which indicates the removal of impurities affecting the crystallization and melting behavior of the material.

On the crystallization thermograms of analyzed samples, only two exothermic peaks were observed, which may be attributed to PP’s very low content and the fact that its crystallization occurred at very similar temperatures to HDPE and led to the overlapping of peaks [62]. Similar effects related to the temperature positions of crystallization peaks of PE and PP were noted by Hajj et al. [63]. It can be seen that the crystallization temperature of both PE types, determined as the temperature position of the peak, is the highest for sample M1, indicating faster crystallization due to the presence of impurities, as suggested by the FTIR analysis. Solid particles may act as a nucleating agent, increasing the crystallization rate [64].

**Figure 8:**
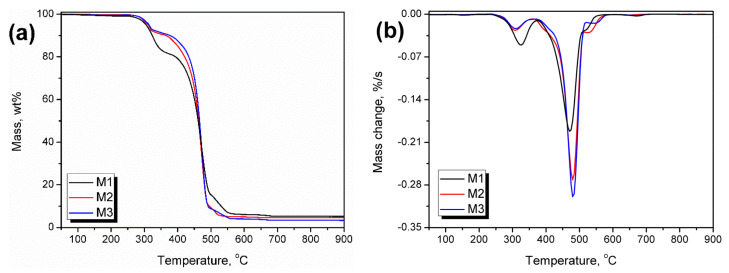
Plots of (**a**) mass change and (**b**) differential thermogravimetric curves of wastes.

The results of the thermogravimetric analysis of investigated wastes are presented in Figure 8 and summarized in Table 3. It can be seen that the washing of materials enhances their thermal stability, which can be attributed to the removal of organic residues, especially plant-based materials, whose stability is significantly lower compared to polyolefins [65]. Literature data indicate that the decomposition of hemicellulose and cellulose occurs in the range of 250–350 °C, which is in line with the temperature position of the first peak on DTG curves (T_max1_) [66]. It can be seen that the washing of wastes results in the drop of this peak’s magnitude, indicating efficient removal of the organic residues. The second peak (T_max2_) was attributed to the polymer phase’s primary decomposition step, both polyethylene, and polypropylene. It can be seen that its magnitude increases for M2 and M3 samples, pointing to the reduction in contaminants’ share. The temperature position of T_max2_ is typical for polyolefins [67]. According to the literature reports, the decomposition of polyolefins occurs between 300 and 500 °C, with the fastest rate around 440–460 °C for PP and 460–475 °C for polyethylene [68,69,70,71]. Generally, the polyolefins are completely degrading, with the residue usually lower than 1.0 wt % [68]. Therefore, another indicator of the washing process’s efficiency is the decrease in char residue, pointing to the decreasing content of mineral impurities, which are often stable above 900 °C.

### 3.2. Compatibilization of Wastes

**Figure 9:**
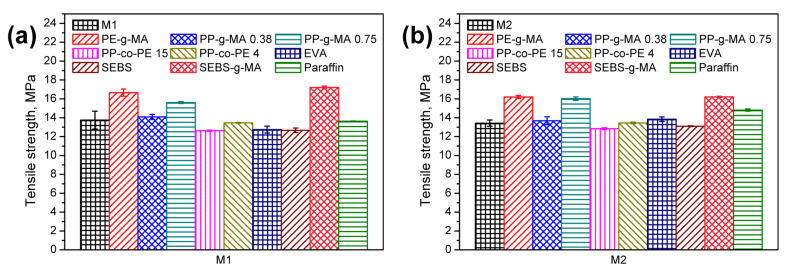

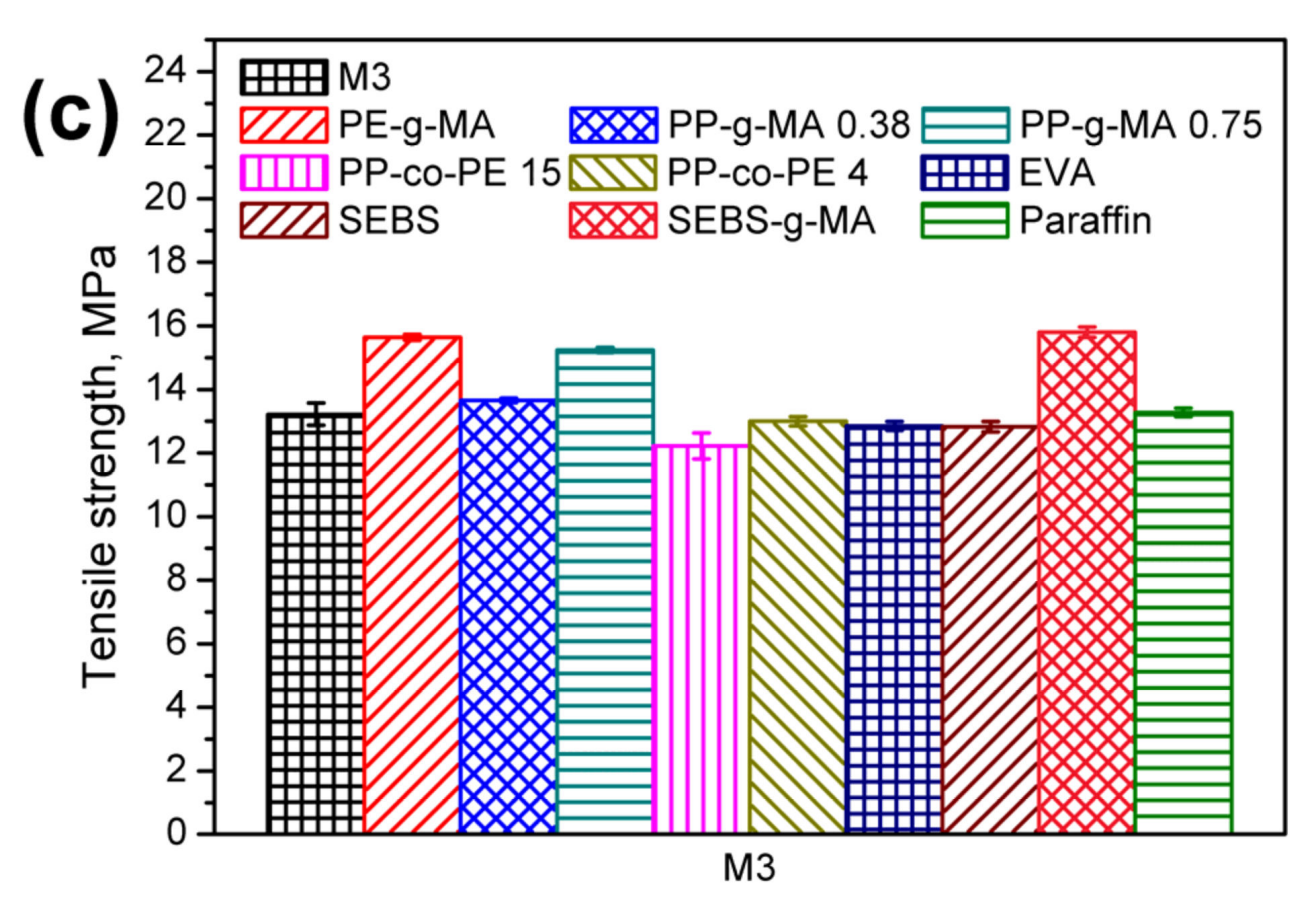
Tensile strength of compatibilized (**a**) M1, (**b**) M2, and (**c**) M3 wastes.

**Figure 10:**
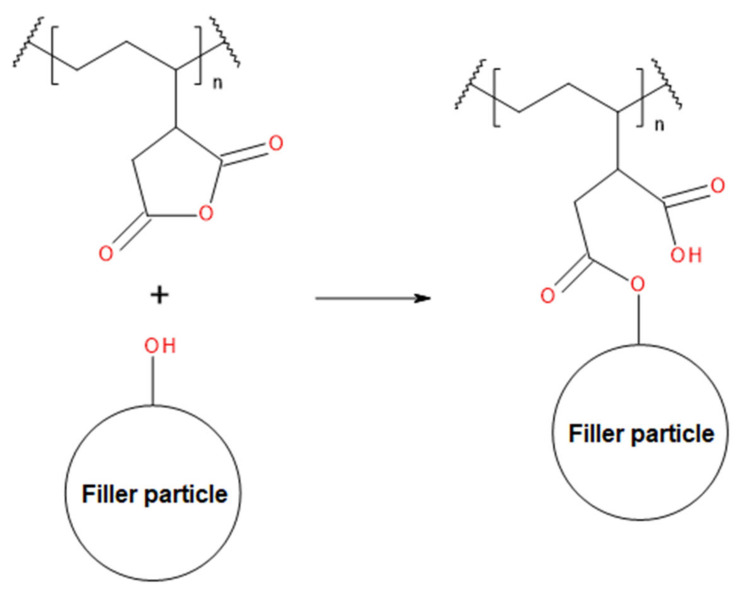
The general scheme of interactions between maleic anhydride and hydroxyl groups present on the surface of filler particles.

In Figure 9, there are presented values of tensile strength of compatibilized wastes. It can be seen that some of the analyzed compatibilizers were found to be very useful in enhancing the tensile strength of materials. The reinforcing effect was especially noted for the compounds with grafted maleic anhydride. It was attributed to the possibility of interfacial covalent bonding provided by the maleic anhydride [72]. The exemplary scheme of these interactions is presented in Figure 10. The beneficial impact of anhydride containing modifiers on the compatibility of PE-based materials was previously reported by other researchers [73].

The beneficial effects of maleic anhydride on polymer composites’ compatibility were repeatedly reported in the literature, both for lignocellulose [74,75] and mineral [76] fillers. Such an effect is associated with the partial “crosslinking” of material due to generation of covalent bonds [77]. In the presented work, the best results were noted for PE-g-MA, SEBS-g-MA, and PP-g-MA. The effect was more significant for grafted polyethylene than polypropylene, attributed to the differences in maleic anhydride content. The applied PE-g-MA compatibilizer contained around 7% of MA, while PP-g-MA contained about ten times less. Therefore, the impact of higher PP strength was neglected. Considering SEBS-g-MA, the reinforcing effect was associated with its higher ductility compared to polyolefins [78]. Despite the lower MA content (1.5%) compared to PE-g-MA, the impact of SEBS-g-MA on tensile performance was similar.

The addition of EVA did not affect the tensile strength significantly, and no reinforcing effect was noted. It was associated with the high vinyl acetate content and more ductile behavior of the applied compatibilizer. The strength of EVA materials is noticeably affected by their composition. An increase in VA content from 11 to 44% may decrease the tensile strength over nine times, simultaneously gradually increasing the ductility [79]. Moreover, EVA may inhibit the crack propagation in polyethylene, enhancing its strength [80].

**Figure 11:**
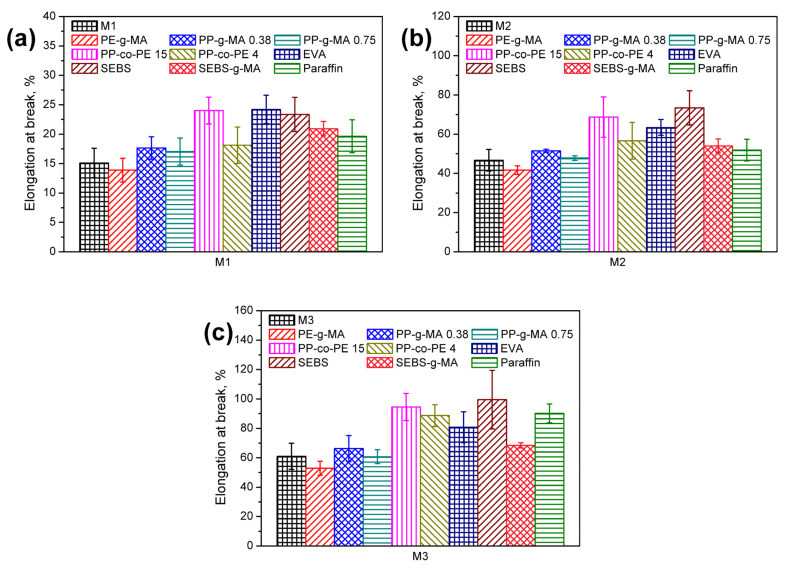
Elongation at break of compatibilized (**a**) M1, (**b**) M2, and (**c**) M3 wastes.

The impact of applied modifiers on the elongation at break of wastes was presented in Figure 11. It can be seen that the best effects were caused by the incorporation of the EVA, SEBS (neat or grafted), and copolymer of PP and PE (especially with higher PE content). Other researchers noted similar effects [81]. Svab et al. [82] noted an almost 50% increase in elongation at break of polypropylene after 5 wt % addition of PP–PE copolymers. The enhancement of polyolefins’ ductility by adding PP-co-PE compatibilizers was also noted by Wang et al. [83]. Chen et al. [84] reported an over 100% increase in the elongation at break of polystyrene/polypropylene blend and composites for the 2 wt % addition of SEBS. When the compatibilizer content was increased to 5 wt %, the elongation was six times higher than for uncompatibilized composite. Additionally, Denac et al. [78] noted the enhancement of PP and PP/talc composites’ elongation by adding SEBS and SEBS-g-MA. In both works, the Authors attributed the compatibilizer’s beneficial impact to the reduced stiffness and increased ductility, which was also indicated by Banerjee et al. [85]. According to Parameswaranpillai et al. [86], this effect can be attributed to the compatibility of ethylene–butadiene blocks in SEBS with the non-crystalline fraction of polyolefins facilitating interfacial diffusion of segments. Gradual increase in elongation at break of the polyolefin-based matrix was also noted by Alanalp and Durmus [87]. Based on their results, relatively low styrene content in the applied compatibilizer (29%) promoted its miscibility with the polyolefin chain enhancing the ductility of the material.

Considering EVA, Pham et al. [88] reported a 21% rise of elongation at break when only 3 wt % of this material was introduced into LDPE. Su et al. [89] found that the EVA addition to PP/LDPE blend was more beneficial towards elongation at break than PP-g-MA, confirming the presented results. In the presented work, the high effectivity of EVA compatibilizer was attributed to the high vinyl acetate content, which implicates the ductility of the material [90].

The ductility of analyzed materials was also noticeably enhanced by the addition of paraffin, indicating the plasticizing effect on the PE matrix. Such an effect was also suggested by Popelka et al. [91].

Contrary to the tensile strength, PE-g-MA was not very efficient in enhancing elongation at break, attributed to the increased stiffness of materials. The “crosslinking” caused by the maleic anhydride reduced the ductility of the material. Nevertheless, for lower content of MA (PP-g-MA compatibilizers), slight enhancement was noted, which confirmed the results of other works [92].

**Figure 12:**
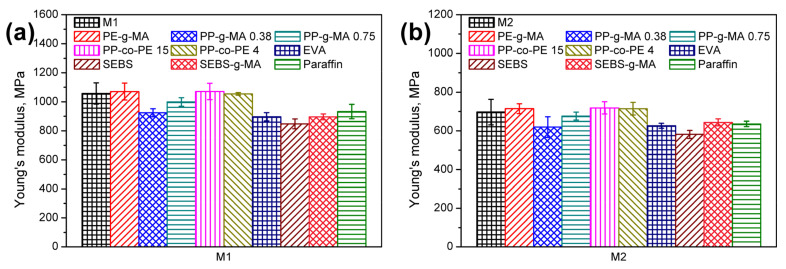

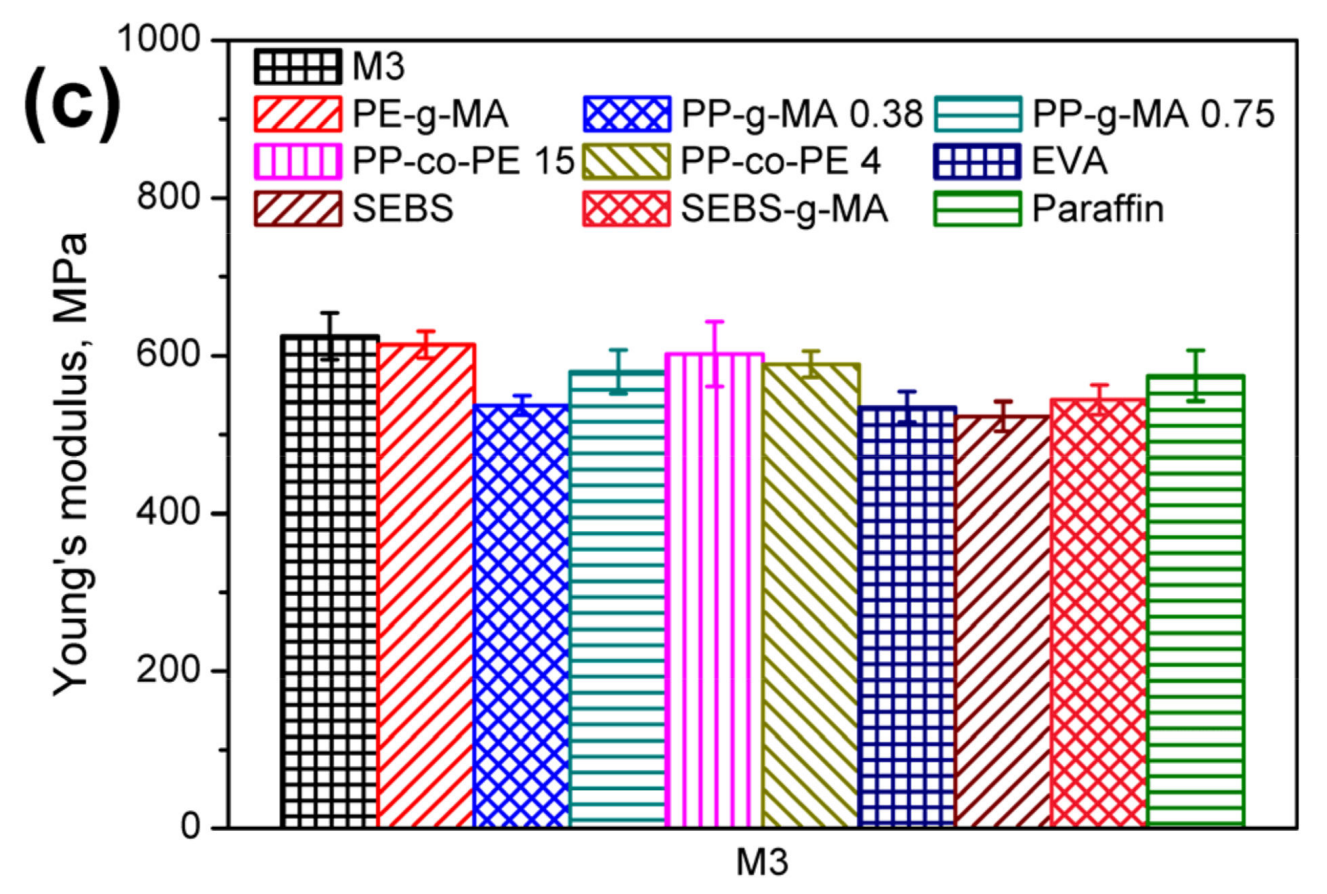
Young’s modulus of compatibilized (**a**) M1, (**b**) M2, and (**c**) M3 wastes.

Figure 12 shows the values of Young’s modulus for compatibilized wastes M1–M3. It can be seen that, generally, the introduction of analyzed modifiers caused a reduction in stiffness of materials. The exceptional performance was noted for PE-g-MA and PP-co-PE materials. In the first case, the noted effect was attributed to the high maleic anhydride content (15%), which noticeably strengthened the interfacial interactions with mineral and lignocellulose solid particles present in wastes. For compatibilizers with lower MA content, a reduction in stiffness was noted. Brito et al. [92] also reported the reduced stiffness of polyethylene filled with low mineral filler contents after the addition of compatibilizer with maleic anhydride. Nevertheless, the enhancement level decreased with each washing cycle, which was associated with this process’s efficiency. For PP-co-PE, the effect was attributed to the stiffness of compatibilizers themselves. The most significant reduction in Young’s modulus was noted for EVA, SEBS, and paraffin compatibilizers. It was associated with enhanced ductility, as suggested by the values of the elongation at break and other literature reports [86,93]. The reduction was observed even when SEBS-g-MA was introduced. Therefore, the enhancement of the interfacial interactions did not overcome the relatively low stiffness of SEBS. Similar effects were noted by Denac et al. [78]. Moreover, they attributed the reduction in stiffness to the changes in crystallization behavior due to maleic anhydride. Elnahas et al. [94] reported the linear decrease in LDPE material’s stiffness with the increasing paraffin content. For 2 wt % addition, Young’s modulus was reduced by 5%, which was attributed to the presence of relatively short hydrocarbon chains compared to the structure of polyethylene. Therefore, paraffin may act as a plasticizer of polyethylene, which was also suggested by Mpanza and Luyt [95].

Similar observations were made by Alothman [96] when 5 wt % of EVA was incorporated into HDPE. Two types of this material, differing in VA content (6.5 and 27.0 wt %) were analyzed. Their introduction caused 5.3 and 9.5% reduction in Young’s modulus, respectively. Over 10% drops were noted in the presented work, but VA content was significantly higher −39%. Changes in materials’ stiffness were attributed to the reduction in their crystallinity.

**Figure 13:**
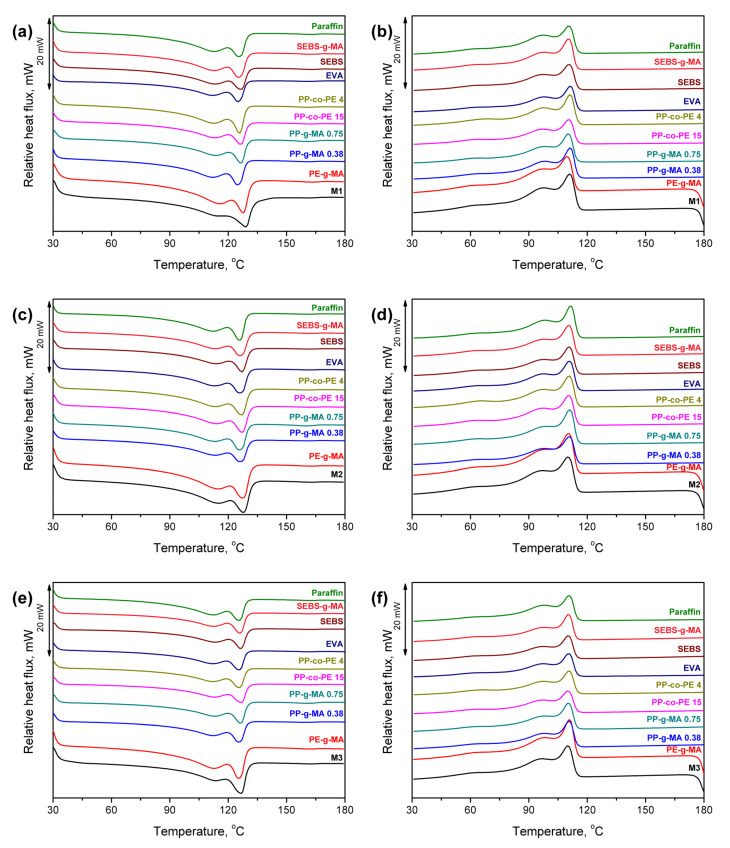
Differential scanning calorimetry thermograms (presented in “exo up” mode) for (**a**,**c**,**e**) heating and (**b**,**d**,**f**) cooling of compatibilized (**a**,**b**) M1, (**c**,**d**) M2 and (**e**,**f**) M3 wastes.

In Figure 13 and Table 4, there are results of the DSC analysis of compatibilized wastes presented. It can be seen that, qualitatively, no significant changes were noted after the addition of analyzed compatibilizers. Some changes were noted in the values of crystallization and melting temperatures. However, they were not very significant, mostly due to the relatively low content of introduced compatibilizers (2.5 wt %). Generally, modifiers’ addition caused the decrease in melting temperatures, pointing to the reduction in crystallite size, accompanied by the reduction in crystallinity degree. The opposite effect was only noted for the PE-g-MA compatibilizer, which is due to the high content of maleic anhydride, which may increase the spherulite size. Similar effects were noted in the work of Lima et al. [97]. Such an effect can also be seen when comparing samples containing SEBS and SEBS-g-MA. However, to a lower extent, because of relatively low MA content compared to the PE-g-MA compatibilizer [98]. Nevertheless, irrespectively of the type of applied SEBS, it slightly reduced the melting temperature of polyethylene phases, which could be attributed to the reduced spherulite size, as suggested by Karger-Kocsis et al. [99]. Dong et al. [100] noted a similar effect, who reported the decrease in PE crystallinity by the inhibition of molecular segment movements. It hindered polyethylene crystallization because macromolecules were not sufficiently arranged, and the grain size distribution was broadened.

The reduction in polyolefin crystallinity after the addition of PP–PE copolymers was also noted by Lu et al. [101]. Interestingly, a slight increase in crystallization temperature was also noted for paraffin addition, which confirms the similar reports of Mpanza and Luyt [102].

The addition of EVA caused a reduction in the polyethylene phase’s melting temperatures due to compatibilizer characteristics and a melting point of applied EVA type (48 °C according to the producer data), which was repeatedly proven by other researchers [79]. Moreover, the crystallinity degree was reduced. It was attributed to the relatively short PE segments suitable for EVA’s crystallization, which affected the PE matrix’s crystallization [103]. In the presented work, the effect of 2.5 wt % EVA addition was quite noticeable due to vinyl acetate’s high content (39%) [96].

**Figure 14:**
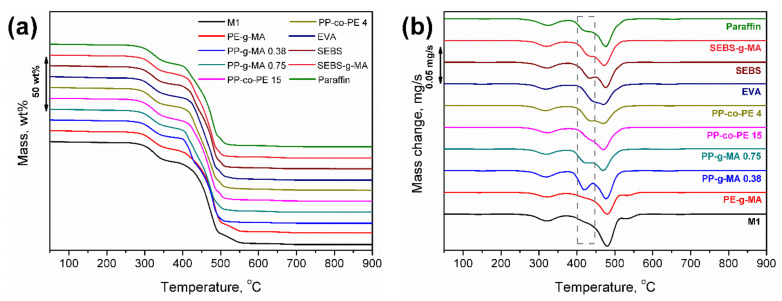
Plots of (**a**) mass change and (**b**) differential thermogravimetric curves of compatibilized M1 wastes.

The results of the thermogravimetric analysis of compatibilized wastes M1–M3 are summarized in Table 5. Moreover, the exemplary curves for the M1 sample are presented in Figure 14. It can be seen that, despite the significant influence of applied modifiers on the mechanical performance of analyzed materials, their impact on thermal decomposition was noticeably lower. Generally, the onset of thermal degradation, determined by the temperature attributed to the 2 wt % mass loss, was hardly affected by the applied compatibilizers. A similar effect was observed for the char residue, which was dependent on the applied waste stream rather than the compatibilizer type.

The most significant differences were noted comparing the course of thermal decomposition, especially the 400–450 °C interval, resulting from the incorporation of other polymers. When PE-g-MA was used, no additional peak was noted. The additional signal was noted for PP-based compatibilizers, which was related to the slightly lower polypropylene stability than polyethylene [67]. The higher values of T_max2_ also expressed this relationship for samples containing PP-co-PE 15 compared to PP-co-PE 4. For EVA addition, the peak intensity was rather low because of the characteristics of its decomposition. It is characterized by two-step decomposition associated with the presence of ethylene and vinyl acetate units [103]. With a maximum rate around 345–350 °C attributed to VA’s degradation, the first step was here overlapped with T_max1_. With a maximum rate of about 460–465 °C, the second step caused a broadening of T_max3_ [104]. Compared to EVA, the T_max2_ peak was noticeably more intense for the addition of SEBS-based compatibilizers. Helal et al. [105] reported the maximum decomposition rate of SEBS with 30% of styrene at 430 °C. Lower styrene content enhances SEBS thermal stability [106], similar to maleic anhydride grafting [107].

## 4. Conclusions and Future Directions of Research

Without the additional modification, the wastes generated during agricultural films’ primary use are suitable for landfilling or utilization in a waste incineration plant. In the presented work, we proposed a simple solution based on the extrusion process, commonly applied in the industry. The developed solution may significantly enhance waste materials’ performance and broaden their range of potential applications. Material recycling represents the most straightforward way of managing plastic waste and, at the same time, it enables attainment of new polymer-based materials which can be reused in other sectors than agriculture. The experimental results showed that, after appropriate treatments, the wastes from the recycling line may be applied as an input into other production processes. The addition of only 2.5 wt % of conventional, commonly available compatibilizers, including maleic anhydride-grafted polyolefins, SEBS block copolymers, EVA copolymer or paraffin wax, may overcome the drawbacks caused by the presence of impurities. Presented results indicate that such modification may significantly enhance the ductility of the material and reduce its stiffness, which could be attributed to its improved homogeneity. The use of compatibilizers containing maleic anhydride caused 13–25% enhancement of tensile strength, which was associated with the enhanced interfacial interactions. The highest level of enhancement was noted for the M1 waste characterized by the highest impurities content. Such an effect confirmed the efficient compatibilization of analyzed materials. All of the applied compatibilizers except PE-g-MA caused the increase in elongation at break, even up to 55–64% for EVA and paraffin. The enhancement of materials’ ductility was confirmed by the drop of Young’s modulus, even up to 20% noted for SEBS and EVA. The inefficiency of the PE-g-MA compatibilizer was attributed to the high content of maleic anhydride and polymer materials’ stiffening. Thermal performance of analyzed wastes was only slightly affected by applied modifications. The slight reduction in melting temperatures of polyethylene phase (up to 2.8 °C) and its crystallinity degree (up to 16%) was noted. For more contaminated materials, the addition of compatibilizers caused a minor reduction in the thermal degradation onset (up to 6 °C). At the same time, for the waste after three washing cycles, thermal stability was improved. Nevertheless, for all materials, thermal stability guarantees the safe processing window. This is one of the most important conclusions: depending on the desired properties and application, materials do not have to go through the whole recycling line, reducing energy and water consumption.

Future research works related to the utilization of waste materials from plastics recycling lines should focus on improving the properties of new waste-based materials and reducing their environmental impacts by possible simplifications of the recycling procedures. The performance enhancement could be obtained by introducing various compatibilizers or other conventionally applied methods for enhancement of plastics’ compatibility. Among them should be mentioned static mixing or melt filtration during the extrusion, which are relatively simple methods, which can efficiently improve the homogeneity of the material. The reduction in the environmental impacts of material could be reached by the optimization of the recycling process and, when possible, by using the materials which do not have to undergo the whole process, as shown in the presented manuscript. Despite the presence of impurities, they could be applied especially in the manufacturing of products which do not require exceptional mechanical performance. Moreover, the process’s actual environmental burdens and different materials generated by the processing of applied wastes should be evaluated by life cycle assessment and other available methods.

## Figures and Tables

**Table 1:** List of compatibilizers applied in presented work.

Trade Name	Abbreviation	Chemical Composition	Producer
Linocene PEMA 4351	PE-g-MA	Maleic anhydride grafted PE (~7% of MA)	Clariant (Muttenz, Switzerland)
Exxelor PO1015	PP-g-MA 0.38	Maleic anhydride grafted PP (0.38% of MA)	ExxonMobil (Irving, TX, USA)
Exxelor PO1020	PP-g-MA 0.75	Maleic anhydride grafted PP (0.75% of MA)	ExxonMobil (Irving, TX, USA)
Vistamaxx 6202	PP-co-PE 15	Isotactic PP/PE copolymer (15% of PE)	ExxonMobil (Irving, TX, USA)
Vistamaxx 3588FL	PP-co-PE 4	Isotactic PP/PE copolymer (4% of PE)	ExxonMobil (Irving, TX, USA)
Escorene UL 5540	EVA	Ethylene-vinyl acetate (39% of VA)	ExxonMobil (Irving, TX, USA)
Taipol 6150	SEBS	SEBS block copolymer (29% of styrene)	Dow (Midland, TX, USA)
Taipol 7126	SEBS-g-MA	Maleic anhydride grafted SEBS (1.5% of MA)	Dow (Midland, TX, USA)
Sarawax SX105	Paraffin	Hard paraffin wax	Shell (Houston, TX, USA)

**Table 2:** Values of carbonyl index calculated for M1-M3 wastes according to the literature data [48,49,50,51,52,53].

**Wavenumber, cm^−1^**	**Sample**		
	**M1**	**M2**	**M3**
	**Absorbance**		
720	0.1948	0.2147	0.2324
1380	0.0697	0.0667	0.0637
1460	0.1666	0.1817	0.1964
1720	0.0298	0.0248	0.0211
**Calculation Method**	**Carbonyl Index**		
Height 1720/720 [48]	0.153	0.116	0.091
Height 1720/1380 [49]	0.428	0.372	0.331
Height 1720/1460 [50]	0.179	0.136	0.107
Area (1850–1650)/(1500–1420) [51]	0.694	0.548	0.425
Area (1850–1630)/1380 [52]	2.640	2.177	1.840
Area (1700–1780)/1460 [53]	0.279	0.225	0.184

**Table 3:** Temperatures of 2, 5, 10, and 50% mass losses, values of residue at 900 °C, and temperature positions of peaks on differential thermogravimetric curves determined for M1-M3 wastes.

Sample	T_−2%_, °C	T_−5%_, °C	T_−10%_, °C	T_−50%_, °C	Residue at 900 °C, wt %	T_max1_, °C	T_max2_, °C
M1	280.0	303.9	322.5	461.4	5.22	324.0	473.7
M2	290.2	309.8	361.5	472.6	4.60	306.5	481.7
M3	292.0	313.8	375.0	475.9	3.35	308.5	483.7

**Table 4:** Temperatures of melting and crystallization of particular phases and their degree of crystallinity determined for compatibilized M1–M3 wastes.

Material	Compatibilizer	T_mLDPE_, °C	T_mHDPE_, °C	X_crPE_, %	T_mPP_, °C	X_crPP_, %	T_crLDPE_, °C	T_crHDPE_, °C
M1	-	114.5	128.4	29.73	162.2	0.61	98.1	111.6
	PE-g-MA	115.2	127.1	30.15	162.6	0.49	98.4	110.3
	PP-g-MA 0.38	111.8	124.2	27.15	159.7	0.51	98.4	111.9
	PP-g-MA 0.75	113.3	126.0	26.42	160.8	0.52	98.5	110.8
	PP-co-PE 15	113.2	126.1	26.06	161.1	0.44	97.3	110.6
	PP-co-PE 4	112.1	125.3	26.27	160.3	0.50	98.0	111.7
	EVA	111.7	124.5	26.94	161.7	0.43	98.1	111.7
	SEBS	113.0	126.1	25.68	161.8	0.51	97.7	111.1
	SEBS-g-MA	112.4	124.7	26.49	161.7	0.48	98.2	111.0
	Paraffin	112.4	125.2	26.70	162.1	0.34	98.0	111.1
M2	-	114.4	127.3	30.99	161.4	0.40	97.8	111.0
	PE-g-MA	114.4	126.9	32.63	161.5	0.38	99.5	111.0
	PP-g-MA 0.38	113.3	125.7	27.92	159.5	0.28	98.5	111.3
	PP-g-MA 0.75	112.8	125.2	27.90	159.3	0.34	99.2	111.6
	PP-co-PE 15	113.6	126.5	28.02	161.9	0.32	97.7	111.2
	PP-co-PE 4	113.3	126.3	27.31	159.3	0.30	97.7	111.2
	EVA	113.0	125.5	26.37	161.0	0.35	97.8	111.3
	SEBS	113.4	126.6	26.22	161.3	0.29	98.1	111.3
	SEBS-g-MA	113.2	125.6	26.80	160.5	0.36	98.3	111.2
	Paraffin	112.0	125.4	28.11	159.5	0.46	98.8	112.2
M3	-	113.5	126.0	25.53	161.4	0.36	97.2	110.5
	PE-g-MA	112.4	124.9	27.06	161.1	0.36	98.9	111.5
	PP-g-MA 0.38	112.2	125.6	21.95	159.3	0.38	98.3	111.1
	PP-g-MA 0.75	112.9	126.1	21.87	161.0	0.40	98.6	110.8
	PP-co-PE 15	112.9	126.3	21.46	161.2	0.29	97.3	110.6
	PP-co-PE 4	111.7	125.3	21.51	161.0	0.28	97.8	111.1
	EVA	112.0	125.3	21.57	161.1	0.36	97.8	110.9
	SEBS	113.1	126.0	21.35	161.9	0.35	97.6	110.7
	SEBS-g-MA	112.4	125.6	21.92	161.7	0.35	97.9	110.9
	Paraffin	112.0	126.2	23.35	160.9	0.36	98.2	111.1

**Table 5:** Temperatures of 2, 5, 10 and 50% mass losses, values of residue at 900 °C and temperature positions of peaks on differential thermogravimetric curves determined for compatibilized M1-M3 wastes.

Material	Compatibilizer	T_−2%_, °C	T_−5%_, °C	T_−10%_, °C	T_−50%_, °C	Residue at 900 °C, wt %	T_max1_, °C	T_max2_, °C	T_max3_, °C
M1	-	280.0	303.9	322.5	461.4	5.22	324.0	-	473.7
	PE-g-MA	279.3	304.7	324.5	468.6	6.03	320.3	-	480.6
	PP-g-MA 0.38	280.5	304.2	324.1	453.5	4.78	321.9	428.0	484.4
	PP-g-MA 0.75	274.5	302.6	322.5	449.3	5.03	322.3	431.7	476.8
	PP-co-PE 15	278.3	302.3	321.7	456.4	5.33	325.8	446.5	478.7
	PP-co-PE 4	279.1	302.0	320.3	451.4	5.39	319.8	441.3	478.3
	EVA	274.3	301.7	320.9	454.4	4.56	321.3	446.2	478.5
	SEBS	276.8	303.2	323.2	457.2	5.10	324.7	442.6	483.7
	SEBS-g-MA	276.3	304.1	323.3	454.8	5.05	322.9	445.2	479.7
	Paraffin	278.8	304.5	324.6	458.9	5.28	328.1	436.3	484.2
M2	-	290.2	309.8	361.5	472.6	4.60	306.5	-	481.7
	PE-g-MA	289.8	307.3	351.8	470.5	4.29	303.1	-	480.8
	PP-g-MA 0.38	288.9	309.8	356.6	458.0	4.77	308.4	440.4	479.6
	PP-g-MA 0.75	285.0	308.3	354.9	451.2	3.69	308.1	440.8	475.8
	PP-co-PE 15	291.1	310.3	356.1	460.6	4.08	307.3	443.0	486.0
	PP-co-PE 4	291.0	312.1	358.3	455.7	3.83	309.8	441.4	481.6
	EVA	289.0	309.2	353.6	457.5	3.79	308.1	433.3	478.8
	SEBS	290.8	305.1	357.1	461.0	3.88	308.5	435.0	481.6
	SEBS-g-MA	292.4	312.1	353.5	459.5	4.12	306.5	437.0	484.5
	Paraffin	289.1	310.5	352.8	461.1	3.80	311.8	436.0	486.3
M3	-	292.0	313.8	375.0	475.9	3.35	308.5	-	483.7
	PE-g-MA	291.9	311.2	371.5	475.0	3.25	308.3	-	484.4
	PP-g-MA 0.38	286.3	310.5	362.0	463.0	3.50	309.1	432.2	479.4
	PP-g-MA 0.75	289.5	312.6	371.4	454.5	2.84	310.1	441.4	487.6
	PP-co-PE 15	292.4	311.5	361.2	465.3	2.86	310.1	442.3	482.7
	PP-co-PE 4	294.0	313.9	372.7	458.0	2.79	310.8	436.1	482.3
	EVA	292.8	311.2	360.9	460.2	2.71	309.5	438.6	483.8
	SEBS	292.2	312.1	365.7	464.7	2.84	309.4	439.5	486.6
	SEBS-g-MA	294.7	312.1	362.3	463.6	3.05	311.3	437.1	482.8
	Paraffin	294.0	313.7	361.1	465.1	2.91	313.2	439.5	488.6

## Data Availability

Data is contained within the article. The data presented in this study are available in Waste streams from agricultural film recycling line as a potential polymer materials.
